# A Membrane-Fusion Model That Exploits a β-to-α Transition in the Hydrophobic Domains of Syntaxin 1A and Synaptobrevin 2

**DOI:** 10.3390/ijms18071582

**Published:** 2017-07-21

**Authors:** Cameron B. Gundersen

**Affiliations:** Department of Molecular & Medical Pharmacology, David Geffen UCLA School of Medicine, Los Angeles, CA 90095, USA; cgundersen@mednet.ucla.edu

**Keywords:** synaptic vesicle, exocytosis, SNARE proteins, secondary-structure transitions, regulated secretion

## Abstract

Parallel zippering of the SNARE domains of syntaxin 1A/B, SNAP-25, and VAMP/synaptobrevin 2 is widely regarded as supplying the driving force for exocytotic events at nerve terminals and elsewhere. However, in spite of intensive research, no consensus has been reached concerning the molecular mechanism by which these SNARE proteins catalyze membrane fusion. As an alternative to SNARE-based models, a scenario was developed in which synaptotagmin 1 (or, 2) can serve as a template to guide lipid movements that underlie fast, synchronous exocytosis at nerve terminals. This “dyad model” advanced a novel proposal concerning the membrane disposition of the palmitoylated, cysteine-rich region of these synaptotagmins. Unexpectedly, it now emerges that a similar principle can be exploited to reveal how the hydrophobic, carboxyl-terminal domains of syntaxin 1A and synaptobrevin 2 can perturb membrane structure at the interface between a docked synaptic vesicle and the plasma membrane. These “β-to-α transition” models will be compared and contrasted with other proposals for how macromolecules are thought to intervene to drive membrane fusion.

## 1. Introduction

The soluble, *N*-ethylmaleimide sensitive factor attachment protein receptor (SNARE) complex has dominated discussions of the molecular machinery of membrane fusion [1,2,3,4]. The observation that neuronal SNARE proteins are specific targets for the proteolytic activity of the clostridial neurotoxins [5], along with evidence that SNAREs can augment the rate of fusion of artificial membranes, in vitro (reviewed in: [6,7,8,9]) has led to systematic efforts to understand the molecular mechanism by which SNARE complex assembly drives the terminal steps of the membrane fusion cascade. In spite of wide-ranging efforts to clarify this process, the field has not converged on a commonly accepted model of SNARE action. This disconnect is exemplified in recent papers which advance distinctly different scenarios by which SNAREs might induce the membrane fusion step underlying fast synchronous exocytosis at nerve terminals [10,11,12,13,14,15,16]. A prominent concern for most of these models is that they are incompatible with a large body of electron microscopic data which reveals that synaptic vesicles directly contact the plasma membrane prior to the fusion event [17]. One exception to this criticism is the model in which a gap-junction-like arrangement of SNAREs (and, possibly, other proteins) forms a fusion pore [14]. However, this model [14] is difficult to reconcile with the organization of proteins at the vesicle-plasma membrane interface at frog motor nerve terminals [18] or with images of filaments affiliated with the area of contact between a synaptic vesicle and the presynaptic plasma membrane of cultured neurons [19]. The clear inference from these observations is that considerable uncertainty persists regarding the molecular events that underlie regulated exocytosis in neurons and other cells.

As a counterpoint to SNARE-based models of synaptic vesicle exocytosis, it was hypothesized that a quartet of synaptotagmins (syts) can provide a template for membrane fusion at nerve terminals [20]. A key feature of this “dyad model” is that it envisioned that the palmitoylated, cysteine-rich region of syt 1 (or, 2) adopts an interfacial, β-strand structure in the synaptic vesicle membrane. Once a vesicle docks at the plasma membrane, this region re-locates to the vesicle-plasma membrane interface and aids in catalyzing membrane fusion. Interestingly, the “β-to-α transition” (or, BAT) fusion models described here begin with a similar assumption: namely, that the carboxyl-terminal, hydrophobic domains of syntaxin 1A (or B; syx) and synaptobrevin 2 (syb; also referred to as VAMP for vesicle-associated membrane protein; [21]) adopt an intra-membrane β-structure prior to SNARE complex formation. The BAT models then postulate that full SNARE zippering leads to structural changes that promote membrane fusion. In the ongoing effort to distinguish among the competing ideas for how fusion events are regulated, the BAT models have attractive features and limitations that will be addressed in the Discussion.

## 2. Description of the BAT Models

### 2.1. The BAT Models: Assumptions

BAT models require several assumptions about the structural properties and protein organization of a “docked and primed” synaptic vesicle. For present purposes, a docked synaptic vesicle is defined as a vesicle whose limiting membrane is in direct contact with the plasma membrane. A primed vesicle belongs to a subset of docked vesicles for which the “SNARE fusion machinery” (as described below) is fully assembled. The assumptions embodied in both BAT models are enumerated here and are followed by a full description of each model.

**Figure 1:**
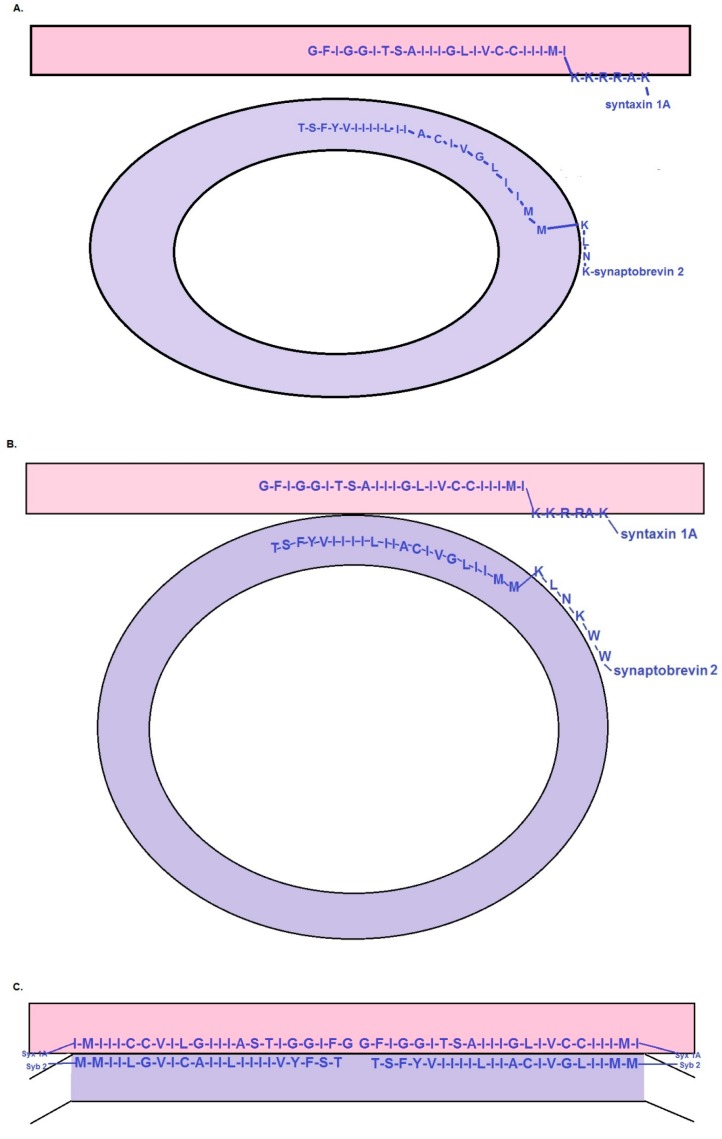

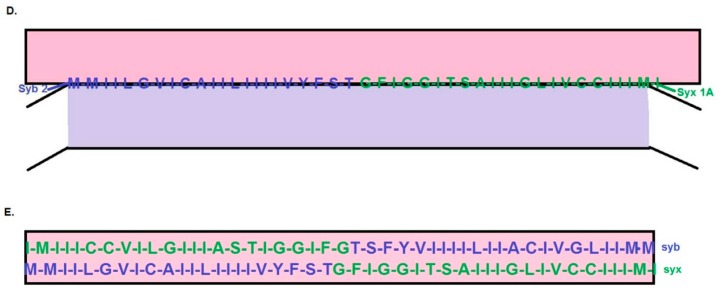
Proposed membrane organization of syb and syx for the BAT models. (**A**) The pre-docking state of a synaptic vesicle (lavender) is shown prior to contact with the plasma membrane (pink). The intra-membrane, hydrophobic domains of vesicle-associated syb and plasma membrane-associated syx (in dark blue with the single letter code denoting the amino acid sequences of the mouse proteins here and in all later panels) are shown at the interface between the inner and outer hemi-bilayers. These intra-membrane sequences are proposed to adopt β-structure which is preserved in panels (**B**–**E**). Palmitoylation of C (cysteine) residues is not depicted. The C-ends of syb and syx are presumably modified to eliminate their charge (**B**) Once SNARE zippering commences and the synaptic vesicle contacts the plasma membrane, it initiates a translocation of the hydrophobic domains of syb and syx from the inter-leaflet position shown here toward the vesicle-plasma membrane interface as illustrated in C (**C**) This shows an intermediate state in which the hydrophobic domains of syb and syx are re-locating to the vesicle-plasma membrane interface (**D**) This is a cross-sectional representation of the docked and primed state of the synaptic vesicle-plasma membrane interface for the BAT model. Syb sequence is dark blue while syx is green. The syx partner for syb would sit behind the plane of this image, as would the syb partner for syx (**E**) “Top” view of the organization of syb-syx pairs of a docked and primed synaptic vesicle. This is the postulated end-to-end organization of the syb-syx pairs that one would observe by removing the external leaflet of the plasma membrane thereby exposing the elongated β-structure of these domains. Note that these images are drawn to accentuate the arrangement of syb and syx, first within their respective membranes and then at the interface between the vesicle and plasmalemma.

**Figure 2:**
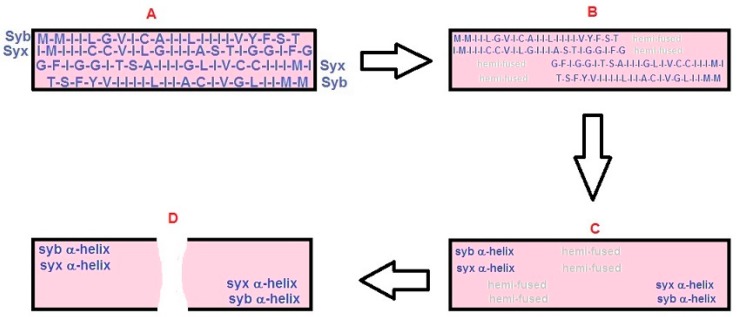
Organization of syb and syx for the short BAT model and the fusion sequence. (**A**) The short BAT model involves the same steps shown in Figure 1A–C, but instead of an end-to-end organization of the inter-membrane domains of syb and syx (as in Figure 1D,E), they reside side-by-side. Shown is a “top” view (that removes the outer hemi-bilayer of the plasma membrane). This is the primed state of the short BAT model (**B**) From the same perspective as in A, this “top” view begins the process that leads to membrane fusion. The inter-membrane segments of syb and syx have begun to retract laterally (denoted by the smaller font size) and this transition from helix leads to hemi-fusion in the areas that had been occupied by protein (**C**) Complete β-to-α transition of the syb and syx inter-membrane regions leads to larger areas of hemi-fusion that culminate in full fusion in (**D**). Possible causes of full fusion are discussed in the text. Color scheme: same as Figure 1.

**Figure 3:**
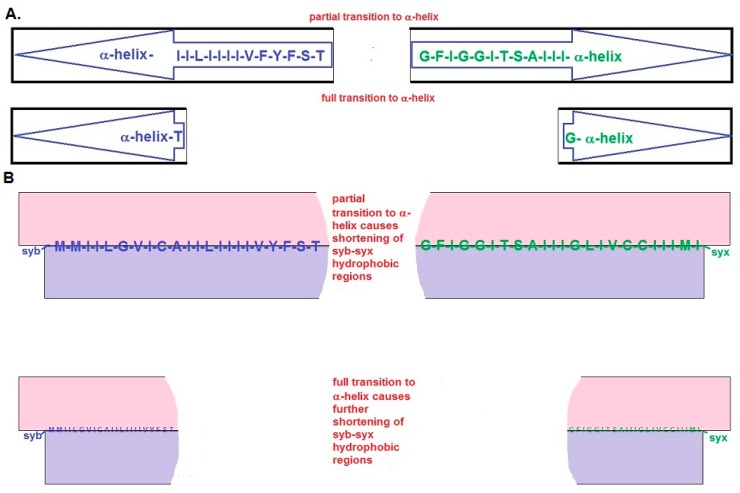

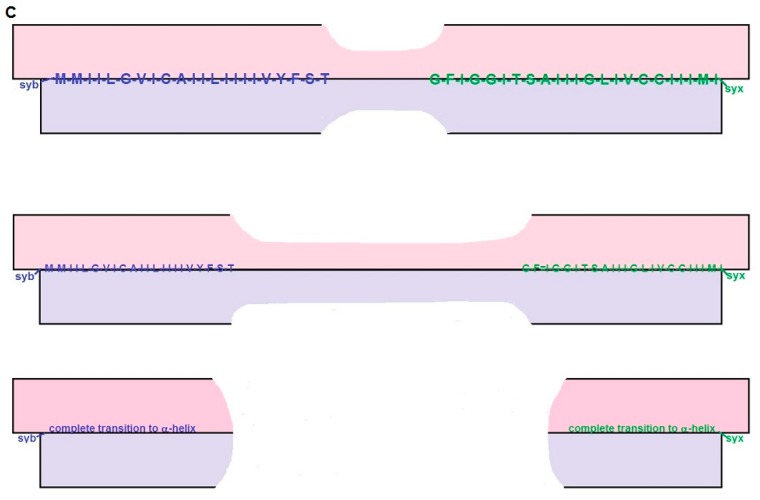
Fusion sequence for the BAT model. (**A**) The upper panel shows a cross-sectional view of a partial transition to α-helix of the syb (in blue) and syx (in green) with a nascent, central fusion pore. The lower panel shows full helical transition of the syb and syx pairs with an expanded fusion pore. In both panels the arrowhead represents the region that has transitioned to helix (**B**) This is a cross-section of the partial and full transition to α-helix which depicts the shortening of the syb (in blue) and syx (in green) and the coating of the ends of these polypeptides by membrane lipids. The shortening is represented by the use of smaller font for the membrane-embedded sequences of syb and syx (**C**) In contrast to the sequence in A&B, the transition to full fusion may include a hemi-fusion intermediate that resolves to full fusion via mechanisms discussed in the text.

(1) Syb and syx hydrophobic domains adopt a β-structure: Prior to synaptic vesicle docking, the carboxyl-terminal, hydrophobic regions of syb and syx are assumed to adopt an intra-membrane β-structure (Figure 1A). This contrasts with virtually all prior SNARE models which envision a helical, transmembrane conformation for these hydrophobic domains. Thus, prior to docking at the plasma membrane, the last 22 residues of syb and the final 23 residues of syx reside at the interface between the inner and outer hemi-bilayers of the vesicular and plasma membranes, respectively (Figure 1A). In Figure 1B, a synaptic vesicle has just made point-like contact with the plasma membrane. The hydrophobic domains of syb and syx still reside within the membrane interior, but as SNARE zippering proceeds, this localization changes (see assumption 2). A corollary to this assumption is that the terminal carboxyl groups of syb and syx are modified to neutralize their negative charge thereby helping to facilitate this intra-membrane localization.

(2) Docking of a synaptic vesicle at the plasma membrane initiates a change in the location of the hydrophobic domains of syb and syt: Ongoing SNARE zippering brings the synaptic vesicle membrane into direct contact with the plasma membrane (Figure 1B). Once contact is made, the hydrophobic β-motifs of syb and syx transit toward the vesicle-plasma membrane interface (Figure 1C). This movement of syb-syx pairs occurs on either side of the zone of vesicle-plasma membrane contact (shown in cross-section in Figure 1C). Although this re-distribution of the hydrophobic domains of syb and syt may be driven by zippering of the SNAREs, it might also require assistance from other “chaperone” proteins. Once both of the syb-syx pairs reach the vesicle-plasma membrane interface (Figure 1D,E; the organization of syb and syx for the short BAT model is in Figure 2A) their polypeptide backbones engage in hydrogen bonding to diminish polarity and stabilize their inter-molecular association.

(3) The pre-fusion, primed state of the BAT models: In the BAT model, a pair of SNARE complexes becomes situated at the vesicle-plasma membrane interface such that the hydrophobic, β-structure regions of syb and syx face one another end-to-end (side view in Figure 1D; top view in Figure 1E). In the short BAT model, the SNARE pairs reside side-by-side (Figure 2A). For both models, SNARE zippering must be arrested external to the zone of membrane contact to prevent α-helical coiling from influencing the β-structure of the intra-membrane, hydrophobic regions of syb and syx.

(4) SNAREs catalyze membrane fusion via physical shortening: The critical assumption of the BAT models is that the dimensions of the interfacial SNARE assemblies change once the arrest of SNARE zippering is relieved. With the resumption of SNARE zippering, the key transition that propagates into the region of the apposed membranes is that the β-structure of the SNARE hydrophobic domains converts to α-helix (Figure 2 and Figure 3A–C). For the BAT model, this “β-to-α” transition causes a rapid shortening of the four, opposed hydrophobic domains of syb and syx (Figure 3A,B) and leads directly (Figure 3B) or indirectly (via hemi-fusion, Figure 3C) to fusion pore formation. In the short BAT model, rapid retraction of the intramembrane domains of the SNARE proteins seeds the formation of parallel hemi-fused domains connected by a central zone of hemi-fusion (Figure 2) which culminates in full fusion. Possible mechanisms driving full fusion in the short BAT scheme are addressed below.

### 2.2. The “BAT” Model: Step-by-Step Description

(1) Docking and priming: The initial steps of the BAT model resemble most SNARE-based models [3,10,22,23]. Specifically, as a synaptic vesicle approaches the active zone, it reaches a distance at which syb can begin to engage with (open) syx and SNAP-25 to initiate the zippering reaction that leads to SNARE complex formation. The initial, partial zippering of the SNARE complex draws the vesicle membrane into direct contact with the plasma membrane (Figure 1A–D). A key demand of the BAT model is that SNARE zippering cannot progress to the point at which it would affect the structure of the hydrophobic regions of syb and syx shown in Figure 1D,E. The BAT model does not predict the point at which the arrest of SNARE zippering occurs, but a reasonable inference is that it precedes the regions identified as charged linkers in the work of Stein and colleagues ([24]; namely, residue 85 for mouse syb2 and residue 256 for mouse syx 1A).

(2) Initiation of the fusion sequence: The BAT model also follows conventional SNARE-based schemes in that it requires a mechanism by which Ca^2+^-bound syt releases the arrest of SNARE zippering, thereby allowing α-helical coiling and coiled-coil interaction of the hydrophobic regions of syb and syx. The BAT model envisions that the rapid transition of these β-motifs to α-helix abruptly shortens these domains. The retraction of these polypeptides leads to two possible outcomes (described in 3 and 4 below).

(3) SNARE hydrophobic domains serve as templates for membrane lipid re-arrangement: The rapid, β-to-α transition of the hydrophobic regions of syb and syx directly nucleates lipid movements to create a fusion pore (Figure 3A,B). The presumption for this scenario is that the lipids of the inner hemi-bilayer of the synaptic vesicle membrane and the outer hemi-bilayer of the plasma membrane curl around and “coat” the exposed carboxyl termini of the abruptly retracting SNARE pairs. Intrinsic shortening of the SNARE pairs dilates the fusion pore. The extent of this shortening can be predicted from established structural data: https://swissmodel.expasy.org/course/text/chapter1.htm. The typical inter-residue distance for a β-sheet is 0.35 nm. Hence, the 23 residues of proposed β-structure of syx should extend ~8.0 nm. By the same token, 3.6 residues contribute to each turn of an α-helix, and one turn covers a distance of 0.54 nm. Full transition to α-helix of the 23 residues of syx will yield a structure ~3.5 nm long. Collectively, these results predict a shortening of ~4.5 nm in each direction. Assuming that the C-termini of the retracting SNARE pairs acquire a lipid “coat” that is up to 3 nm wide, this yields an initial fusion pore diameter of ~3 nm.

(4) Retraction of the SNARE hydrophobic domains causes the formation of a hemi-fusion intermediate: In this variation (Figure 3C), the helical transition of the intra-membranous SNARE pairs leads to transient annealing between the outer hemi-bilayer of the plasma membrane and the inner hemi-bilayer of the synaptic vesicle. The progression from the hemi-fused state to full fusion presumably is triggered by changes in the lipid state in the hemi-fusion diaphragm and by other factors. These factors may include the permeability of this diaphragm, hydrostatic pressure of the vesicle contents, and the effects of the intrinsic tension of the highly curved vesicle membrane. Another consideration is that as the hydrophobic domains of syb and syx transition from β-to-α, the combined effects of hydrostatic influences and vesicle membrane tension will be to induce an extracellularly directed splaying of the nascent protein helices. This action may also help to drive the formation and expansion of the fusion pore. Finally, as the helical transition progresses toward the C-terminus, the palmitoylated cysteine residues of syb and syx will retract and briefly rotate from their inter-laminar position in a manner that is likely to induce a local perturbation of bilayer structure that may aid fusion.

### 2.3. The Short BAT Model: Step-by-Step Description

The short BAT model starts with the same assumptions as the BAT model. However, once a synaptic vesicle docks at the plasma membrane, the hydrophobic domains of the syb-syx pairs align side-by-side (Figure 2A) rather than end-to-end (as in Figure 1D,E). With this organization, the short BAT scenario predicts a smaller interface between a docked/primed synaptic vesicle and the plasma membrane than in the BAT model. However, as outlined below, the membrane perturbations triggered by the β-to-α transition of these hydrophobic domains provides a less-obvious path for driving membrane fusion than in the BAT model.

As with the BAT model, the short BAT model envisions the resumption of SNARE zippering leading to a retraction of the dual SNARE pairs within the zone of synaptic vesicle-plasma membrane contact. Partial retraction is shown in Figure 2B with full retraction in Figure 2C. These intra-membrane movements lead to linear zones of membrane hemi-fusion that form parallel to each retracting SNARE-pair (hemi-fused zones are in gray in Figure 2B,C). Once the SNARE pairs have fully retracted (from ~8 nm to ~3.5 nm), there will be a central, ~1nm wide region of hemi-fused membrane, orthogonally connecting the two parallel hemi-fused zones. The short BAT model assumes that some combination of the factors enumerated above (step 4 of the BAT model) leads to full fusion.

## 3. Discussion

The goal of the BAT models is to explain how SNARE proteins could influence structural transitions at the interface between a synaptic vesicle and the plasma membrane, or more-generally, a trafficking organelle and its target membrane. A shared feature of these schemes is that they conform to abundant evidence pointing to SNAREs as vital elements for driving membrane fusion in cells as diverse as yeast and human neurons [3,10,12,13,22,23,25]. Nevertheless, BAT models contain assumptions and make predictions that are appreciably different from the dyad hypothesis and other SNARE-based models of membrane fusion. Consequently, ongoing empirical work will be necessary to distinguish among these scenarios and bring us closer to an understanding of the molecular events that mediate and regulate biological membrane fusion.

Three key experimental tests will define the future prospects of the BAT models. First, the structure of the hydrophobic regions of syb and syx (as proposed in Figure 1A) needs to be determined in a bilayer membrane environment prior to SNARE complex assembly. Second, it will be necessary to test whether β-structural elements are present at the synaptic vesicle-plasma membrane interface as predicted in Figure 1C or Figure 2A. Third, the BAT models require that the C-termini of syb and syx be charge-neutralized. Although strategies are available to acquire data germane to these issues, the ensuing discussion addresses considerations that will be necessary to ensure that the experimental outcomes are not misleading.

Seminal models of SNARE complex formation [1,2,3] depicted syb and syx as proteins tethered to their respective membranes via transmembrane anchors oriented perpendicular to the plane of the membrane. In the model of Hanson and colleagues [3], zippering of the SNARE domains was proposed to bring a synaptic vesicle progressively closer to the plasma membrane in a sequence that could lead to membrane fusion. Later, structural evidence that SNARE zippering proceeds through the “linker” regions that connect the SNARE domains of syb and syx to their (α-helical) hydrophobic tails [24] strengthened the idea that full SNARE zippering might be a central step in the fusion cascade. Although these data appear to be inconsistent with the BAT models, this is not the case: First, the structural data of Stein and colleagues [24] addressed the “end state” of SNARE zippering. The BAT models argue that the propagated transition of β-structure to α-helix drives membrane fusion. Thus, the “end state” of each BAT model is exactly the state of the hydrophobic regions of syb and syx reported by Stein and colleagues [24]. Instead, the crucial question concerns the secondary structure of these regions *prior* to the initiation of SNARE zippering. Thus, structural determinations need to be performed by using syb and syx under conditions that mimic, as closely as possible, their native environment before SNARE zippering begins. These efforts need to use versions of syb and syx that reflect their in vivo state. In this latter context, the most important consideration is the evidence that syb and syx are fatty acylated on the cysteine residues in their hydrophobic domains [26,27]. In the case of the cysteine residues in the juxta-membrane region of syts 1 and 2, it was argued that covalently linked palmitoyl moieties are likely to be important structurally for stabilizing the proposed β-strand of these motifs [20]. By analogy, palmitoylation of the lone cysteine residue of syb and the tandem cysteines of syx may be equally important structural determinants. Concurrently, it was suggested that post-translational modifications that eliminate the charge on the C-terminal residues of syb and syx are important to facilitate their integration into the membrane interior as proposed in Figure 1A. Thus, it will be interesting to determine whether C-end modifications are associated with native syb and syx and influence the secondary structure and membrane organization of these proteins.

Since their discovery, syb [21,28] and syx [29] have been regarded as being anchored to membranes via hydrophobic, C-terminal helical motifs. Structural analyses have been somewhat equivocal regarding the conformation of these hydrophobic domains: Circular dichroism measurements and infrared spectrometry indicated that the intra-membrane domain of syb was largely α-helical with a prominent tilt relative to the plane of the membrane [30]. However, these experiments of Bowen and Brunger [30] also detected significant levels of extended β-structure and anti-parallel β-sheet in the hydrophobic domain of syb. Combinations of α and β-structure were also observed for peptides based on the hydrophobic domains of syb and syx [31]. Subsequent NMR analyses favored a helical conformation for the intra-membrane domain of syb embedded in dodecylphosphocholine micelles [32]. However, this last investigation also noted that the evidence supporting a helical propensity for the intra-membrane region relied on data from soluble proteins which may not be directly applicable to hydrophobic motifs. Finally, an issue for all of these studies is that they used constructs that were not palmitoylated and this modification may influence the secondary structure of these domains, in vivo.

Based on the preceding observations, it is reasonable to ask whether there is a more-compelling rationale for proposing that the hydrophobic domains of syb and syx adopt β-structure in biological membranes. Support for this idea comes from the Biological Magnetic Resonance Data Bank (http://www.bmrb.wisc.edu/referenc/choufas.shtml), which tabulates the secondary structure preferences of amino acid residues. Application of these propensity values to the 22 C-terminal residues of mouse syb 2 and the 23 C-end residues of mouse syx 1A, yields the following insights: for syb 2 the mean helical propensity of the hydrophobic region is 1.03. This corresponds to a weak likelihood of α-helix formation. At the same time, the average β propensity for this region is 1.36, a value corresponding to a strong β former. For syx 1A, the average helical propensity is 0.94 which is on the cusp of a weak-to-indifferent helix former. The β propensity for this region is 1.33 which is solidly within the range for β-strand formation. Thus, average propensities favor β-structure (note that these calculations do not take into account palmitoylation of the cysteine residues). Looked at from another angle, there are only four residues in syb 2 and three in syx 1A for which the preference for helix exceeds the preference for β strand. Further, both syb 2 and syx 1A have “helix-breaking”, glycine residues within their C-end regions (see sequences in Figure 1A; note that the glycine in syb 2 has been suggested to induce a pre-fusion kink [33]), which also diminishes the likelihood that these domains natively adopt helix. Taken together, these considerations strengthen the conclusion that new empirical data will be crucial to clarify the bilayer membrane structure of these regions of syb and syx prior to full SNARE zippering.

In the context of the preceding discussion, it is of interest that mutations of the hydrophobic domain of syb 2 that favor helix formation suppress exocytosis and impair fusion pore expansion in chromaffin cells [34]. Conversely, mutations favoring β-structure restore exocytosis and accelerate fusion-pore expansion. These results were interpreted as being indicative of a conformational interplay between membrane lipids and the helical backbone of the transmembrane domain of syb 2 [34]. However, these results also fit within the framework of the BAT models, because mutations that stabilize the helical state of the hydrophobic region of syb 2 should preclude the adoption of the state represented in Figure 1C,D and Figure 2A. At the same time, enhancing the β-character of this region should support membrane fusion, as long as the β-to-α transition triggered by full SNARE zippering is not abridged.

The preceding work [34] also speaks to the general question of the functional significance of the intra-membrane, hydrophobic domains of SNAREs. This remains an issue of considerable controversy, and Dhara and colleagues [34] review the discordant results obtained by several other groups. Because the BAT models depend on conformational changes of the C-terminal hydrophobic domains of syb and syx, future studies of the functional contribution of these motifs will help to determine the validity of the BAT proposals and resolve questions about the role of these motifs.

Mutagenesis experiments have also targeted the C-terminus of syb 2 [35]. Addition of tandem lysines, histidines, or glutamates failed to support the recovery of exocytosis in chromaffin cells from syb2-cellubrevin double knockout mice. Pairs of glutamine residues also prevented the recovery of exocytosis, while single lysine or glutamate residues at the C-terminus enabled an ~20% recovery of exocytosis. Exocytotic recovery was partial with pairs of valine residues and complete with glycine pairs. Overall, these results are compatible with BAT models which depend on the ability of the C-ends of syb and syx to adopt an intra-membrane configuration. One caveat concerns the modest exocytotic recovery seen with single, C-end lysine or glutamate residues. These results may reflect post-translational modification of these residues that eliminates their charge, but does not support membrane fusion as well as the native residues.

It was argued [17] that recent electron microscopic investigations of the synaptic vesicle-plasma membrane interface constrain models of fast, synchronous exocytosis at nerve terminals. Two key observations were identified. First, “docking filaments” were reported to traverse the zone of contact between a synaptic vesicle and the plasma membrane [19]. Second, the work of Jung and colleagues [36] revealed that synaptic vesicles with the greatest area of contact with the plasma membrane were depleted during synaptic activity. This latter finding was interpreted as indicating that vesicles with large contact areas had the highest probability of undergoing exocytosis. Are the BAT models compatible with these findings? To begin, Cole and colleagues [19] inferred that docking filaments were likely to include SNAREpins. BAT models provide a straightforward explanation for how SNARE proteins could be organized at this interface without triggering concerns about the prominent surface charge profile of assembled SNARE complexes [17]. However, further work clearly is needed to identify these filaments. The BAT models also predict the dimensions of the synaptic vesicle-plasma membrane contact zone: By using an inter-residue distance of 0.35 nm per amino acid residue in a β-sheet and assuming that the ends of the SNARE pairs touch one another (as in Figure 1D,E) or project across the contact area (Figure 2A), the hydrophobic regions of the SNAREs will traverse a contact zone of ~16 nm in the BAT model and ~8 nm in the short BAT version. If these contact zones were circular, their areas would be ~200 and 50 nm^2^, respectively. For purposes of comparison, the dyad model estimated this area of contact at 70–80 nm^2^ [20]. All of these areas are appreciably smaller than the >600 nm^2^ contacts reported by Jung and colleagues [36]. Thus, additional work will be needed to determine whether these large contact zones are present at other synapses, and whether docked and primed synaptic vesicles have proteins within this contact area. 

Measurements made at the frog neuromuscular junction also revealed that the combined thickness of the vesicular and plasma membranes within their zone of direct contact was exactly twice the thickness of the individual membranes away from the area of contact [36]. An important inference from this result is that nothing is “sandwiched” between the synaptic vesicle and plasma membrane within their region of contact. On the other hand, if something is sandwiched in this area, it does not change the thickness of the contact zone. Applying these considerations to the BAT models leads to an interesting prediction. Specifically, once the hydrophobic domains of syb and syx reach the vesicle-plasma membrane interface (as in Figure 1C–E and Figure 2A), the amino acid side chains of these proteins effectively replace bilayer lipids within this area of the opposed membranes. However, because the amino acid side chains tend to be appreciably shorter than the end-to-end dimension of a cholesterol molecule or a typical membrane phospholipid [37], it is likely that the lipid composition of the outer hemi-bilayer of the plasma membrane and inner hemi-bilayer of the synaptic vesicle is modified both to stabilize this interface and to preserve membrane thickness. To achieve this outcome, at least two local modifications of lipid composition are plausible: First, the lipids in the inner hemi-bilayer of the synaptic vesicle and outer hemi-bilayer of the plasma membrane are likely to be “larger” (that is, a greater distance between their hydrophilic head group and hydrophobic, fatty acyl “tails”). Second, because of its small head group, it is also possible that cholesterol molecules intercalate with the β-strands of syx and syb to stabilize the opposed hemi-bilayers of the synaptic vesicle and plasma membrane. While these considerations may be related to the accumulating evidence that lipid composition influences membrane fusion [37], it will be necessary empirically to clarify whether these predictions are borne out, in vivo.

A recent study of frog active zones reported vesicle-plasma membrane hemi-fusion [36]. These observations extended earlier findings [38], and reprised the question whether hemi-fusion is a bona fide intermediate in the fusion sequence at nerve terminals. This issue was reviewed recently [15], and in the BAT models (Figure 2 and Figure 3), hemi-fusion can be a step in the pathway to full fusion. However, further research will be needed to determine the physiological relevance of hemi-fusion.

Synaptic vesicle exocytosis can occur within 50 microseconds of the rise of cytosolic Ca^2+^ at mammalian nerve terminals [39]. Are the BAT models fast enough to explain this process? The first consideration in answering this question is that BAT models depend on the same reactions (binding of Ca^2+^ to syt followed by full SNARE zippering) that are intrinsic to other SNARE models of exocytosis. Although these steps are unlikely to be rate limiting, they have been criticized for other reasons [10]. These criticisms clearly also pertain to the BAT models. Beyond these steps, the main temporal concern for the BAT models involves the β-to-α transition. Although the kinetics of SNARE zippering have been measured [40,41,42], these efforts have not used constructs containing the last ~20 residues of syb and syx that are relevant to BAT models. However, protein secondary-structure transitions can occur extremely rapidly, and α-helical folding of a 20 amino acid motif can take place in the sub-microsecond range [43]. In the membrane milieu envisioned for the C-termini of syb and syx (Figure 1D,E), even if this conformational change were 20-fold slower, it would be compatible with the kinetics of exocytosis at mammalian synapses. Thus, BAT models can be fast enough to explain rapid, synaptic vesicle exocytosis.

Although several of the preceding considerations reflect favorably on the BAT models, it is useful to compare and contrast features of the BAT models with the dyad hypothesis and other SNARE-based fusion scenarios. The BAT models share with other SNARE models the requirement that SNARE zippering be halted at an intermediate stage. It also requires that Ca^2+^-bound syt act to relieve this arrest of SNARE zippering. Currently, there is no consensus for how these steps occur at the nerve terminals [10,11,12,13,14,16], but there has been abundant interest in the contribution of SNARE-interacting proteins, including the complexins, munc-13 and munc-18 [12,13,23,44]. However, the exact molecular cascade to which these proteins contribute remains unclear. In this respect, a unique feature of the dyad model [20] is that it dispenses with any direct role for SNAREs in the membrane-fusion reaction, and circumvents the need for complexins, munc-13 or munc-18 to participate in the final steps of the fusion sequence. At the same time, a shared feature of the dyad scheme and the BAT model is that they require synaptic vesicles to be in direct contact with the plasma membrane (morphologically docked). In this respect, these models diverge from most other SNARE-based models which begin with synaptic vesicles that do not directly contact the plasma membrane [10,11,12,13,15,16]. It was noted in a recent commentary [17] that most electron microscopic studies of nerve terminals describe a population of (“docked”) synaptic vesicles which directly contact the plasma membrane. At the same time, the work of Jung and colleagues [36] suggested that these vesicles include the release-ready pool. The primary exception to this generalization comes from cryo-electron tomographic studies of synaptosomes in which few docked vesicles are seen [45]. However, a prominent concern for these latter efforts is that the process of synaptosome preparation and sample processing may have depleted docked and/or primed synaptic vesicles and under-represented this population. Thus, further work will be necessary to clarify the ultrastructure and protein organization of docked and primed synaptic vesicles.

The BAT models envision that the highly charged 10 residue “linker” region (as identified in [24]) preceding the postulated β-structural domains of syb and syx resides external to the junction where the synaptic vesicle membrane contacts the plasma membrane. In fact, this region can be considered as playing an “anchoring” role during the structural transitions proposed in Figs. 2 and 3. This anchoring function reflects the challenge of accommodating this region within the hydrophobic interior of the juxtaposed membranes. However, in some SNARE-based fusion models [11,14], this highly charged region appears to become compressed between the vesicular and plasma membranes. Achieving this compression presumably demands a mechanism for charge neutralization that is not specified in these models. Nevertheless, structural studies [30] and simulations [33] suggest that the linker region of syb can be partially subsumed within the membrane interior. At the same time, in vitro studies indicate that the linker region of syb is likely to be appreciably less important for the overall fusion cascade than the amino acid residues at the C-end of the SNARE domain which were critical for proteoliposome fusion [46]. Independently, it was observed that the specific nature of the membrane association of syntaxin 1a profoundly influenced its interaction with other proteins, like syb and synaptotagmin [47]. Consequently, further work will be needed to understand the contributions of the syb linker and the hydrophobic regions of syb and syx to the fusion cascade.

An important goal of the BAT models is to explain the fast, synchronous exocytotic events observed at nerve terminals. However, the BAT mechanism may also operate for other forms of constitutive or regulated exocytosis. In fact, given the evidence for multiple molecular pathways for catalyzing membrane fusion [48], it is conceivable that evolution has led to more than one solution for mediating membrane fusion in neurons and other secretory cells. In this context, the disparate predictions embodied in the BAT model, the dyad model [20], and other schemes [11,12,13,14,15,16] should be amenable to empirical analysis and lead to a better understanding of the molecular events that underlie the fusion of biological membranes.

## Abbreviations

Syb	Synaptobrevin
Syx	Syntaxin
SNAP-25	Synaptosome-associated protein of 25 kDa
SNARE	Soluble, *N*-ethylmaleimide sensitive factor attachment protein receptor
BAT	β-to-α transition
